# NeuroRAD-FM: A Distributionally Robust Foundation Model for Precision Neuro-Oncology

**DOI:** 10.21203/rs.3.rs-10655143/v1

**Published:** 2026-09-08

**Authors:** Moinak Bhattacharya, Annie Singh, Angelica P. Kurtz, Fabio M. Iwamoto, Prateek Prasanna, Gagandeep Singh

**Affiliations:** Columbia University Irving Medical Center; Atal Bihari Vajpayee Institute of Medical Sciences; Columbia University Irving Medical Center; Stanford University; Stony Brook University; Columbia University Irving Medical Center

## Abstract

Foundation models for neuro-oncology have shown promise for non-invasive molecular characterization and prognostication, yet their clinical utility remains limited by cross-institutional distribution shift and poor performance on under-represented molecular alterations. We developed NeuroRAD-FM, combining self-supervised pretraining, Group-DRO, and a multi-backbone ensemble to learn site-invariant imaging representations. NeuroRAD-FM is pretrained on 7,414 multi-sequence brain MRI examinations aggregated from multiple institutions and evaluated on independent glioma cohorts from UCSF (n = 111), UPenn (n = 95), and an in-house cohort (n = 292). Downstream tasks included automated tumor segmentation, molecular biomarker prediction, and overall survival prediction. Compared with conventional foundation models, NeuroRAD-FM learned more site-invariant features, improved tumor segmentation across tumor types, significantly enhanced molecular marker prediction, and increased concordance indices across all institutions. These findings suggest that integrating Group-DRO with a multi-backbone foundation model enables robust, site-invariant representation learning and substantially improves molecular subtype and survival prediction across diverse neuro-oncology cohorts.

## Introduction

Brain tumors represent a significant global health challenge, contributing substantially to cancer-related morbidity and mortality. According to recent estimates, primary brain and central nervous system (CNS) tumors account for approximately 1.4% of all cancers worldwide but are responsible for a disproportionately high burden of disease due to their aggressive nature and poor prognosis^1,2^. Specifically, malignant brain tumors such as glioblastoma (GBM) exhibit a dismal five-year survival rate of less than 10%, despite advances in surgical techniques, chemotherapy, and radiotherapy^3–5^. Even lower-grade gliomas, which initially present a more indolent course, eventually progress to higher grades, severely impacting long-term outcomes^6,7^. Accurate diagnosis, prognostication, and molecular characterization are therefore critical for guiding clinical decision-making.

Magnetic Resonance Imaging (MRI) is central to brain tumor evaluation because its multi-sequence acquisitions capture complementary aspects of tissue biology, including tumor morphology, edema, necrosis, and infiltrative margins. Traditional radiomics approaches extract hand-crafted features from segmented tumor regions to predict molecular markers, treatment response, and survival^8,9^. However, radiomics is limited by its dependence on accurate tumor segmentation, sensitivity to acquisition heterogeneity, and restricted ability to capture complex biological patterns^10^. Deep learning addresses some of these limitations by learning hierarchical representations directly from data, with convolutional and transformer-based architectures achieving state-of-the-art performance across medical imaging tasks^11^. Nonetheless, fully supervised models require large, well-annotated datasets and often generalize poorly across heterogeneous clinical environments.

Recent advances in deep learning have enabled foundation models (FMs) that leverage large-scale diverse datasets, to learn transferable representations adaptable to multiple downstream tasks with minimal labeled data^12^. Large-scale pretraining efforts, including MedSAM^13^, MedBLIP^14^, RadImageNet^15^, and RadFM^16^ have demonstrated the promise of foundation modeling for medical imaging. Despite this progress, development of radiology-specific foundation models remains relatively limited^17–19^. The primary reason being lack of sufficient large-scale training data and robust cross-institutional generalization. To avoid these limitations, we use a diverse multi-institutional MRI dataset, leading to a unified representation able to generalize across machines, locations, and medical applications.

Although emerging MRI-based foundation models have shown utility in neurodevelopmental and neurodegenerative applications^20,21^, neuro-oncology applications remain largely unexplored. Many are trained on homogeneous datasets or predominantly healthy brains and fail to generalize across the variability of real-world MRI, including differences in scanners, imaging protocols, and patient populations^19^. Most also depend on tumor segmentation or highly curated datasets that are rarely available in routine practice. As a result, performance often degrades across institutions, while the lack of a unified representation necessitates multiple task-specific models, limiting scalability and clinical adoption.

A major additional barrier is site variance, the pronounced distribution shift introduced by differences in acquisition protocols across institutions. In multi-center datasets, MRI scans often cluster by site rather than tumor biology, leading models to overfit site-specific signatures and fail on unseen hospitals or outlier imaging conditions. This issue is especially problematic for uncommon molecular alterations, where data are inherently limited and heavily imbalanced, underscoring the need for neuro-oncologic models that explicitly address site variability. This motivates the development of a distributionally robust optimization (DRO) framework to learn domain-invariant representations and maintain performance under distributional shift^22^. Existing self-supervised learning methods, including contrastive, reconstruction-based, and self-distillation approaches, learn representations from unlabeled data but generally optimize average-case objectives that fail to account for inter-site heterogeneity^23,24^. Our Group-DRO framework optimizes worst-case performance across imaging sites to learn more domain-invariant representations under distributional shift.

Building on these insights, we introduce NeuroRAD-FM, a neuro-oncology-focused MRI foundation model. Using multi-sequence MRI from diverse institutions, NeuroRAD-FM learns tumor-centric representations without requiring manual segmentation during pretraining. Our DRO-based strategy explicitly accounts for site variability, enabling strong out-of-distribution generalization and efficient adaptation to downstream tasks, including prediction of common and underrepresented molecular biomarkers, overall survival, and multicompartment tumor segmentation. Unlike existing foundation models that rely on a single self-supervised backbone, NeuroRAD-FM pretrains a family of complementary self-supervised learning (SSL) encoders on the same multi-institutional corpus and integrates them through a weighted ensemble. This design preserves the complementary inductive biases of contrastive, reconstruction, and self-distillation objectives, while DRO ensures that each backbone and therefore the ensemble remains robust to site and class imbalance. Together, these capabilities position NeuroRAD-FM as a scalable framework for robust and clinically deployable neuro-oncologic imaging Artificial Intelligence (AI).

## Results

### Patient Characteristics

The foundation model is trained on 7,414 multi-institutional brain tumor MRI scans and independently tested on 498 adult glioma patients from UCSF, UPenn, and an in-house cohort (Fig. 1a). Our in-house cohort from Columbia University Irving Medical Center consisted of 292 patients, of whom 56.8% were male. Most patients were Isocitrate dehydrogenase (IDH) wildtype (87.3%), and O6-methylguanine-DNA methyltransferase (MGMT) promoter methylation was present in 29.5%, while 58.9% were unmethylated and 7.9% partially methylated. Among additional molecular markers, Telomerase reverse transcriptase (TERT) promoter mutation, epidermal growth factor receptor (EGFR), and Cyclin dependent kinase inhibitor 2A/2B (CDKN2A/2B) loss were observed in a subset of tumors, and Tumor protein p53 (TP53) wildtype-like patterns predominated. High proliferative activity was common, with Ki-67 proliferation ≥ 20% observed in the majority of patients (Table 1). Additionally, we use two publicly available datasets from UCSF and UPenn. The UCSF cohort comprised 61.26% male patients, and the UPenn cohort comprised 65.26% male patients. More details on the molecular subtypes are provided in Table 2.

### Distributionally robust foundation model training

We employed a self-supervised neuro-oncology foundation model utilizing all four MRI sequences from the training cohort (Fig. 1b). The encoder model is a 3D ResNet architecture that learns generalizable volumetric representations for predicting different heterogeneous downstream neuro-oncology tasks. To eliminate any risks arising from distributional shifts between organizations, imaging protocols, and disease subtypes, the encoder was optimized using Group Distributionally Robust Optimization (Group-DRO) (Fig. 1c). Instead of averaging the loss on training samples, Group-DRO function relies on the important minority group and trains this model based on its requirements, which ensures the learning of tumor-dependent imaging results while being independent of any cohort-specific characteristics. A more detailed description of the algorithm can be found in the Methods section.

To identify the impact of various self-supervised objectives, four different complementary encoders based on MoCo^25^ (E1), DINO^26^ (E2), MAE^27^ (E3), and BYOL^28^ (E4) algorithms were pre-trained. The learned embeddings from these encoders was combined using weighted ensemble (Fig. 1d), while the weights were calculated from the validation results gained during model building and fixed prior to external testing. This ensemble leverages complementary representation learning strategies to produce more stable and robust feature embeddings across diverse neuro-oncology tasks.

### Learning site-invariant representations through Group-DRO

We assessed whether Group-DRO pretraining produced site-invariant and biologically meaningful representations using Principal Component Analysis (PCA) and (Uniform Manifold Approximation and Projection) UMAP visualization (Fig. 2a) together with silhouette score analysis (Fig. 2b). The effect of Group-DRO on site clustering was model dependent rather than uniformly consistent across all self-supervised learning frameworks. Group-DRO reduced silhouette scores for BYOL (0.292 to 0.046), MAE (0.302 to 0.284) and MoCo (0.447 to 0.437), consistent with reduced clustering by acquisition site. The magnitude of improvement varied substantially across models, with the largest reduction observed for BYOL. In contrast, DINO exhibited increased site separability following Group-DRO pretraining, with the silhouette score increasing from 0.142 to 0.304. However, the silhouette scores were significantly different from those of the existing self-supervised methods, indicating that our proposed method effectively reduces site-specific clustering variability.

Importantly, reducing site variability did not compromise disease separability. As shown in Fig. 2c, the baseline self-supervised methods do not exhibit clear separation between disease groups. In contrast, our proposed method preserves site invariance while producing distinct disease-specific clusters. Specifically, the centroids corresponding to IDH mutant vs. wildtype and MGMT methylated vs. unmethylated samples are clearly separated, demonstrating improved disease discriminability. For the underrepresented genomic markers, Fig. 2d shows the class centroids, where we observe clear separation between the different marker labels. These results suggest that our method maintains disease-relevant biological distinctions while effectively minimizing site-specific variability.

### Foundation model representations improve downstream tumor segmentation

To determine whether segmentation-free pretraining transfers to dense voxel-level prediction, NeuroRAD-FM was evaluated on independent glioma, meningioma and metastasis segmentation benchmarks. Despite never requiring tumor masks during foundation pretraining, NeuroRAD-FM achieved superior segmentation accuracy across every evaluated tumor compartment. For adult glioma patients, NeuroRAD-FM consistently outperformed BrainIAC across all tumor subregions (Fig. 3a). For the enhancing tumor (ET), NeuroRAD-FM achieved a DSC of 83.59 (95% CI: 82.33–84.85) and an IoU of 75.59 (95% CI: 74.26–76.92), outperforming BrainIAC (DSC: 60.8; IoU: 49.0). Similarly, for peritumoral edema (PE), NeuroRAD-FM achieved a DSC of 82.48 (95% CI: 81.51–83.44) and an IoU of 72.66 (95% CI: 71.51–73.81), substantially exceeding BrainIAC (DSC: 43.4; IoU: 30.0). Accurate delineation of the enhancing tumor and peritumoral edema is particularly important for surgical planning, as these regions guide the definition of resection boundaries while balancing maximal tumor removal with preservation of eloquent brain tissue. For the necrotic core (NC), NeuroRAD-FM achieved a DSC of 73.81 (95% CI: 72.04–75.58) and an IoU of 65.24 (95% CI: 63.42–67.06) compared with BrainIAC (DSC: 47.1; IoU: 36.6). Performance improvements extended to necrotic tumor core segmentation, suggesting that the latent representations capture intratumoral heterogeneity beyond simple tumor localization. Representative qualitative examples demonstrated closer agreement with expert annotations across both glioma and meningioma cases (Fig. 3b).

On the BraTS Meningioma dataset, NeuroRAD-FM similarly demonstrated superior segmentation performance across all evaluated tumor compartments, achieving higher DSC and IoU scores than BrainIAC for both ET (DSC: 85.68 (95% CI: 84.16–87.20) vs. 70.64) and PE (DSC: 59.08 (95% CI: 56.26–61.91) vs. 29.43). For the BraTS Metastasis dataset, NeuroRAD-FM also outperformed BrainIAC, achieving a DSC of 58.22 ± 35.25 for the ET region, compared with 38.2 for the baseline model. Similarly, for the tumor core (TC), which is a combination of the ET and NC regions, NeuroRAD-FM achieved a DSC of 60.44 ± 33.32, compared with 45.78 for BrainIAC.

Representative qualitative examples (Fig. 3b) further illustrate that NeuroRAD-FM produced segmentation masks with closer agreement to the ground truth across both glioma and meningioma cases pre-treatment. Representative metastatic cases from both pre- and post-treatment scans are also shown. In both settings, NeuroRAD-FM accurately delineated the enhancing tumor and the surrounding peritumoral edema, producing segmentation masks that closely matched the ground truth.

### NeuroRAD-FM enables robust molecular characterization across institutions

We evaluated the effect of incorporating the Group-DRO loss into standard self-supervised foundation models and ensembling the feature embeddings for predicting key molecular alterations. In Fig. 4a, we compare our proposed method, NeuroRAD-FM, with existing public baselines like MedBLIP2^14^, BioMedCLIP^29^ and BrainIAC^19^ on combined multi-institutional cohorts (UCSF, UPenn, In-house). NeuroRAD-FM consistently outperformed the baselines in the classification of IDH1 mutation status (AUC- NeuroRAD-FM: 85.58 95% CI: 84.34–86.82, BrainIAC: 62.42 95% CI: 61.06–63.78, p < 0.001), and MGMT promoter methylation (AUC- NeuroRAD-FM: 75.59 95% CI: 74.23–76.96, BrainIAC: 65.36 95% CI: 64.16–66.56, p < 0.001). Similarly, for 1p/19q, NeuroRAD-FM outperforms BrainIAC on the combined cohort from UCSF and In-house datasets (AUC- NeuroRAD-FM: 89.29 95% CI: 75.74–99.90, BrainIAC: 82.09 95% CI: 76.28–87.79, p < 0.001). We further visualized the learned foundation model feature representations using box plots for IDH1 mutation status, MGMT promoter methylation, and 1p/19q codeletion, demonstrating that the feature distributions are significantly different between the corresponding molecular classes (IDH-mutant vs. IDH-wildtype, MGMT-methylated vs. unmethylated, and 1p/19q codeleted vs. non-codeleted), highlighting the discriminative nature of the learned embeddings (Fig. 4c). Also, NeuroRAD-FM demonstrated consistent performance across multiple validation cohorts for both IDH1 and MGMT prediction tasks, indicating improved robustness and cross-institutional generalization (Fig. 4d).

### Improved prediction of under-represented molecular alterations

For the under-represented molecular markers, the performance of the NeuroRAD-FM was the strongest and most consistent (Fig. 4b). For CDKN2A/2B homozygous deletion prediction, the AUC values are 80.33 (95% CI: 73.94–86.73) and 65.00 (95% CI: 55.61–74.39) for NeuroRAD-FM and the baseline BrainIAC model respectively, the improvement was statistically significant (p = 0.0032). Similarly, for EGFR amplification, NeuroRAD-FM demonstrated a significant increase in mean AUC relative to the baseline for EGFR (NeuroRAD-FM : 63.86 95% CI: 60.68–67.05, BrainIAC: 56.12 95% CI: 53.20–59.05, p < 0.001), TERT promoter mutation (NeuroRAD-FM : 80.20 (95% CI: 73.16–87.24), BrainIAC: 69.20 95% CI: 62.29–76.11, p = 0.023), TP53 mutation (NeuroRAD-FM : 64.11 95% CI: 61.10-67.12, BrainIAC: 58.58 95% CI: 55.59–61.56, p = 0.008), and Ki-67 index (NeuroRAD-FM : 52.26 95% CI: 49.16–55.36, BrainIAC: 45.47 95% CI: 42.23–48.72, p = 0.001). To further characterize the learned feature representations, we present one-dimensional kernel density estimation (1D KDE) plots of the foundation model embeddings for CDKN2A/2B (homozygous deletion vs. no deletion), EGFR amplification (positive vs. negative), and TERT promoter mutation (positive vs. negative), demonstrating clear separation between molecular classes (Fig. 4e). Additionally, Fig. 4f presents 2D-KDE visualizations of the learned feature distributions for TP53 (wildtype, mutant, and equivocal) and Ki-67 proliferation index (< 10%, 10–19%, and ≥ 20%), further illustrating the ability of NeuroRAD-FM embeddings to capture biologically meaningful molecular differences.

### Foundation representations improve prognostic modeling

In Table 3, we compare the survival prediction performance of NeuroRAD-FM with the baseline model across three independent institutions. NeuroRAD-FM consistently achieved higher concordance indices (c-index) than BrainIAC, improving from 58.40 ± 17.13 to 66.42 ± 6.62 (HR: 1.357 ± 0.321) on the UCSF cohort, from 66.86 ± 5.42 to 69.31 ± 3.21 (HR: 4.266 ± 5.730) on the UPenn cohort, and from 59.11 ± 1.88 to 62.96 ± 5.11 (HR: 1.568 ± 0.304) on the In-house cohort. Additional KM curves for the high- and low-risk patients for the common and under-represented markers are shown in Supplementary Figs. 2 and 3. These results demonstrate consistent improvements in prognostic performance across diverse institutional datasets, highlighting the robustness and generalizability of NeuroRAD-FM for survival prediction.

### Model interpretability supports biological plausibility

To examine the clinical and anatomical plausibility of the foundation model’s predictions, we evaluated the spatial correspondence between the model’s attention regions and the tumor regions across the glioma, metastasis, and meningioma cohorts. Specifically, we quantified the distance (in voxels) between the center of the predicted attention region and the tumor center (Fig. 5a), where smaller distances indicate that the model focuses on anatomically relevant tumor tissue. We further assessed the proportion of attention center regions, derived using 5% and 1% Grad-CAM thresholds, that overlapped with the tumor region (Supplementary Fig. 4). Higher overlap indicates that the model’s most discriminative features are concentrated within the tumor rather than surrounding normal tissue, supporting the anatomical plausibility and clinical relevance of the model’s predictions across all three tumor types. We also performed interpretability analyses using Grad-CAM visualizations on different patients from the internal dataset (Fig. 5b and Supplementary Fig. 5). Four representative cases with the corresponding uncommon molecular alterations ATRX loss, TP53 mutant, 1p/19q codeletion and CDKN2A/2B loss. These visualizations reveal focused attention on tumor regions, providing qualitative evidence that the learned representations capture biologically meaningful and spatially relevant imaging features.

## Discussion

The rapid progress of large-scale foundation models raises an important question: how general should a generalized model be? In natural images, generalization improves when models learn to ignore shortcuts such as texture or background. In medicine and especially oncology, many features that appear shortcut-like actually carry causal or clinically meaningful information^30^. Demographic patterns, treatment effects, anatomic context, and disease-specific biology vary systematically across patient populations and institutions, making blanket invariance undesirable and potentially harmful^30,31^. Brain tumors, in particular, exhibit radiographic signatures shaped by the blood–brain barrier, white-matter architecture, and treatment-induced phenomena such as pseudo-response, which are absent in extracranial cancers^32^. Consequently, a universal oncology foundation model risks failing to capture organ-specific patterns of growth, invasion, and therapy response that are critical for clinical decision-making. Recent work highlights the importance of robustness-aware training in medical foundation models, demonstrating that models trained with distribution-aware objectives exhibit improved generalization across institutions^33,34^. Our findings align with this paradigm, extending it to neuro-oncology and showing that distributionally robust optimization can mitigate site-specific biases in MRI while preserving biologically meaningful tumor representations.

Neuro-oncology–specific foundation models therefore offer a necessary balance: they generalize across scanners, protocols, institutions, and intracranial tumor types, while preserving the biological and imaging constraints unique to intracranial disease, enabling more faithful representation learning and clinically actionable predictions. This balance motivates two design choices that distinguish NeuroRAD-FM from existing radiology foundation models: a Group-DRO objective that acts on the loss rather than the encoder, so it can be combined with any self-supervised backbone, and a multi-backbone weighted ensemble whose per-encoder weights are fixed once via hyperparameter optimization on held-out validation cohorts and then locked at inference. Together these choices target the two failure modes that organ-agnostic and single-backbone neuro foundation models share: site- and class-imbalance bias in the objective, and brittle dependence on the inductive bias of a single encoder family.

Our approach addresses four key limitations of prior methods and jointly differentiates NeuroRAD-FM from organ-agnostic medical foundation models (e.g., MedSAM, MedBLIP, RadFM, RadImageNet) and from single-backbone neuro foundation models. First, by removing the need for manual segmentation during pretraining, NeuroRAD-FM enables large-scale representation learning across heterogeneous MRI datasets, overcoming a major bottleneck in radiomics and supervised CNN pipelines. Grad-CAM–based analyses show that the model consistently attends to tumor and peritumoral regions despite the absence of segmentation supervision (Fig. 5). Second, incorporating DRO explicitly accounts for site and protocol variability, promoting improved robustness to domain-specific variation in several backbone architectures, a critical need in neuro-oncology given inter-institutional differences in scanner hardware, field strength, and acquisition protocols. Third, self-supervised learning reduces dependence on large, labeled datasets and supports efficient finetuning for downstream tasks, including prediction of common biomarkers (MGMT, IDH1, 1p/19q), underrepresented alterations (EGFR, CDKN2A/2B, TERT, TP53, Ki-67), and overall survival, all from a single unified latent representation rather than from a collection of task-specific networks. Fourth, in contrast to neuro foundation models that commit to a single self-supervised backbone, NeuroRAD-FM pretrains complementary encoders on the same multi-institutional corpus and combines them through a weighted ensemble whose per-encoder weights are determined once via hyperparameter optimization on validation cohorts and then locked, so inference is fully deterministic and does not depend on input-dependent gating. This design preserves the complementary inductive biases of contrastive, reconstruction, and self-distillation objectives, lets the ensemble compensate when an individual backbone’s site invariance degrades under DRO (as observed for BYOL and DINO), and provides a structural advantage over single-backbone neuro foundation models that no amount of additional pretraining on a single architecture can recover.

Despite several advantages, a few limitations remain. The retrospective study design increases susceptibility to unmeasured confounding, including variation in treatment timing, intensity, and imaging schedules, which may affect survival estimation. For under-represented biomarkers, class imbalance and small sample sizes reduce statistical power and performance stability. While reduced site separability provides an initial robustness assessment, more granular validation stratified by vendor, protocol, and scanner is needed.

Future work will prioritize prospective, pre-registered multi-center validation to ensure reproducibility and clinical readiness. Calibration analysis and decision-curve evaluation will be essential for assessing clinical utility. In parallel, expanding the framework toward temporal and interventional modeling, as well as multimodal fusion with pathology, genomics, and longitudinal clinical data, may enable integration into digital-twin–based precision neuro-oncology workflows.

In conjunction with DRO and a weighted multi-backbone ensemble, NeuroRAD-FM produces more site-invariant, task-generalizable representations that enhance prediction of both common and underrepresented molecular markers. Although the magnitude of improvement varied across tasks and backbone architectures, the framework demonstrated stable multi-center performance while maintaining prognostic discrimination. With prospective validation, improved calibration, and longitudinal extensions, such models have the potential to advance precision neuro-oncology and strengthen patient stratification for clinical trials.

## Materials and methods

### Study design and overview

This study was approved by the Institutional Review Board (IRB) of the participating institution. The in-house set was collected retrospectively, and patient consent was therefore waived. This study aims to develop a neuro-oncology-specific foundation model using multi-sequence MRI for downstream tasks such as molecular marker prediction and survival analysis. Pre- and post-operative MRI scans with T1N, T1C, T2W, and T2F sequences were collected from publicly available multi-institutional datasets. To guarantee uniformity across institutions, MRI data were standardized using skull stripping, bias correction, registration, resampling, and intensity normalization. For the downstream clinical tasks, MGMT, IDH1, 1p/19q, and EGFR, TERT, CDKN2A/2B, Ki-67, TP53, and overall survival (OS) data were collected from the institutional cohort.

### Study Population

The foundation model training dataset consists of 7,414 MRI scans (including longitudinal follow-up scans) of patients with brain tumors from various multi-institutional sources, including the BraTS^8,35–39^, MU-Glioma-Post^40^, and LUMEIRE^41^ datasets (Supplementary Table 1). We used an independent multi-institutional cohort for testing, which included adult glioma patients from the publicly available University of California, San Francisco (UCSF)^42^ (n = 111), the University of Pennsylvania (UPenn)^43^ (n = 95), and an in-house institutional cohort (n = 292). All patients have complete molecular and clinical data, such as MGMT promoter methylation status, IDH1 mutation, and OS outcomes. For UCSF and the in-house dataset, 1p/19q status information is also available. Furthermore, for the in-house dataset, we have additional marker information like EGFR, CDKN2A/2B, TERT, Ki-67, and TP53. This diverse validation cohort enables robust performance analyses on these downstream tasks. Supplementary Fig. 1 displays the overview of the study cohorts and data splits used for model training and testing.

### Foundation model training

Foundation model training was conducted using all four MR sequences from the training dataset. The model architecture is a 3D ResNet encoder, trained using a self-supervised learning framework (shown in Fig. 1b). To address distributional shifts across institutions and disease subtypes, we employed a Group-DRO loss, which encourages the model to learn representations that are robust across different subgroups by partitioning training samples into predefined groups (e.g., imaging site or cohort) and optimizing the worst-performing group rather than the average loss across all samples min_θ_ max_g∈G_ E(x, y) ∼ g[ℓ_θ_(x, y)]. The Group-DRO loss is designed to promote learning of representations that are less sensitive to site-specific biases and more robust across heterogeneous clinical domains, thereby improving generalization across downstream neuro-oncology tasks from multiple institutions (Fig. 1c). The loss implements worst-group risk minimization by computing a per-patient sample self-supervised loss and aggregating losses within each cohort. Finally, the model converges by backpropagating only the maximum group-wise mean loss, promoting invariant tumor-related features shared across institutions.

We trained four established self-supervised learning methods: MoCo^25^ (E1), DINO^26^ (E2), MAE^27^ (E3), and BYOL^28^ (E4). Their embeddings were integrated using a weighted ensembling strategy (Σ w_i_ ∗ E_i_) that aggregates the embeddings of several models (E_i_) weighted by factors of w_i_ (Fig. 1d). The weights (w_i_) were determined based on proportional mean AUC of each model during validation across diverse downstream tasks. Because ensemble weights were determined entirely during model development and fixed before external evaluation, the reported multi-center performance represents a true external assessment and is not influenced by downstream test data. This ensemble produces more stable and clinically robust features across a variety of scenarios by lowering prediction variance and utilizing the complementary strengths of various architectures.

### Automated Tumor Segmentation

For the tumor segmentation task, the Group-DRO-pretrained 3D ResNet-50 encoder was used to initialize the feature extraction backbone of the segmentation model. During finetuning, the pretrained encoder weights were kept frozen to preserve the robust domain-invariant representations learned during self-supervised pretraining, while a task-specific decoder was trained to predict voxel-wise tumor segmentations (Fig. 1e). The decoder progressively upsampled encoder features through convolutional layers and skip connections to recover spatial information and predict tumor sub-regions, including enhancing tumor, tumor necrotic core, and edema/infiltrating tumor. The training was performed on the BraTS Adult Glioma and Meningioma datasets in a 5-fold cross-validation setting.

### Molecular subtyping framework

Using the ensembled foundation model-extracted image embeddings, we trained a fully supervised classifier to predict the various molecular markers. The corresponding embedding vectors for each patient were L2-normalized and then trained using a multi-layer classifier head consisting of linear layers, Batch Normalization (BatchNorm), ReLU activations, and Dropout. We addressed class imbalance by computing fold-specific class weights and incorporating them into the cross-entropy loss during training. Each fold was trained using PyTorch Lightning with AdamW optimization and early stopping of 20 epochs. For benchmarking, we compared our approach against publicly available foundation models, including MedBLIP2, BioMedCLIP, and BrainIAC, using identical evaluation protocols. All models were trained for 100 epochs with a learning rate of 1e-5 on an NVIDIA A6000 GPU (52 GB VRAM).

### Survival analysis framework

We performed survival prediction using the image embeddings extracted from the ensemble foundation model with clinical time-to-event data (days to death or censoring). Each embedding was passed through a multilayer MLP survival head with LayerNorm, ReLU activations, and Dropout, producing a scalar log-risk estimate. Model training optimized the Cox partial likelihood loss, implemented with dynamic risk set ordering to correctly compute cumulative hazards. The training paradigm is similar to the molecular subtyping experiments.

### Statistical analysis

Model performance was evaluated across multiple downstream tasks, including tumor segmentation, molecular marker prediction, and overall survival analysis. Segmentation performance was assessed using Dice similarity coefficient (DSC) and Intersection-over-Union (IoU). For binary classification tasks, performance was assessed using the area under the receiver operating characteristic curve (AUC). For survival prediction, the concordance index (c-index) was used to measure the agreement between predicted risk scores and actual survival outcomes and statistical comparisons between models were performed using paired bootstrap resampling of concordance indices across test samples. p-values for survival analysis were computed using paired comparisons of concordance indices across cross-validation folds using the Wilcoxon signed-rank test with a significance threshold set at p < 0.05. All analyses were conducted using Python (v3.9) with Scikit-learn, Lifelines, and SciPy libraries.

## Supplementary Material

This is a list of supplementary files associated with this preprint. Click to download.

- neuroradfmappendix.docx

## Figures and Tables

**Figure 1:**
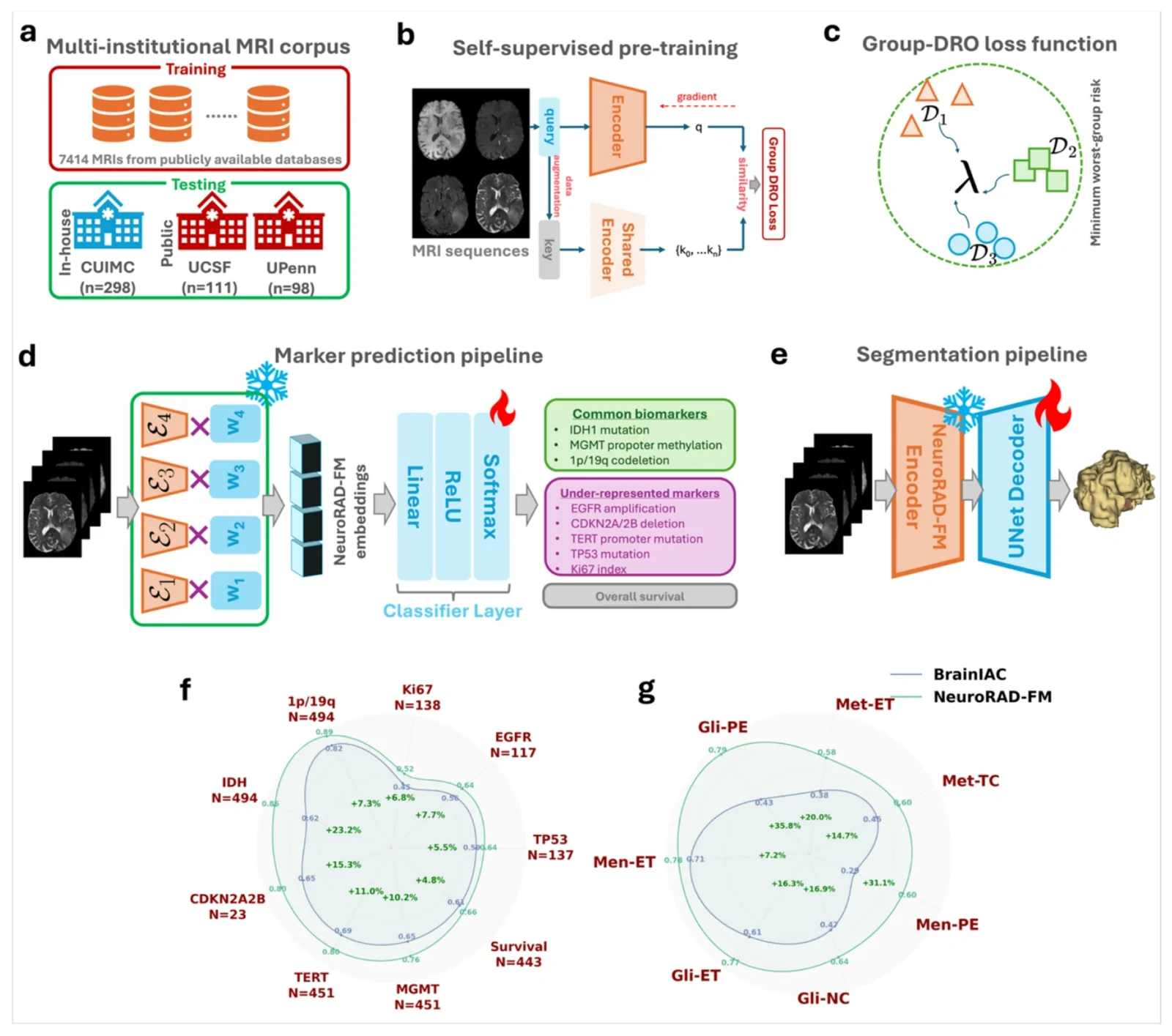
(a) Overview of the training and validation datasets, (b) Pipeline: The proposed framework for training the foundation model in a self-supervised manner, (c) Group-DRO loss, where D_i_ are the group distributions and λ is the group mean, (d) Ensembling and finetuning the pretrained encoders for downstream tasks, (e) Tumor segmentation architecture, (f) Summary of the molecular markers and survival analysis results for NeuroRAD-FM (Ours) and BrainIAC, and (g) Summary of tumor segmentation results (ET: enhancing tumor, NC: necrotic core, PE: peri-tumoral edema, and TC: tumor core).

**Figure 2:**
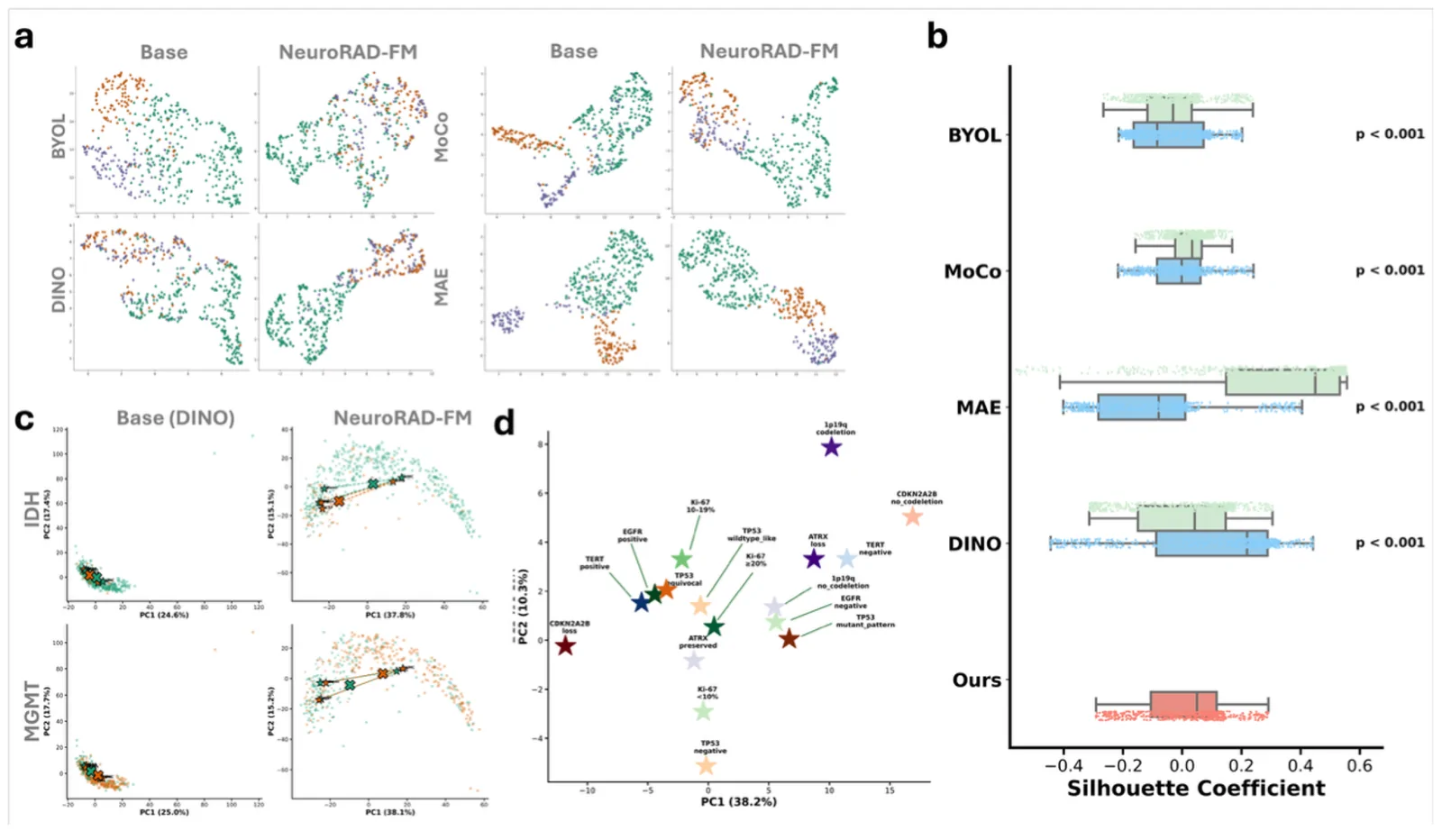
(a) UMAP plots for different SSL methods, (b) Silhouette score for the different Base methods (light green), Base+DRO (sky blue), and ensembled Base+DRO (Ours) methods for robustness estimation, (c) Site-wise (star) and molecular subtype–specific (cross) cluster centroids (IDH-mutant vs wildtype, MGMT-methylated vs unmethylated) for base and NeuroRAD-FM, and (d) Cluster centroids for under-represented molecular subtypes, highlighting the effect of DRO on rare disease representation.

**Figure 3:**
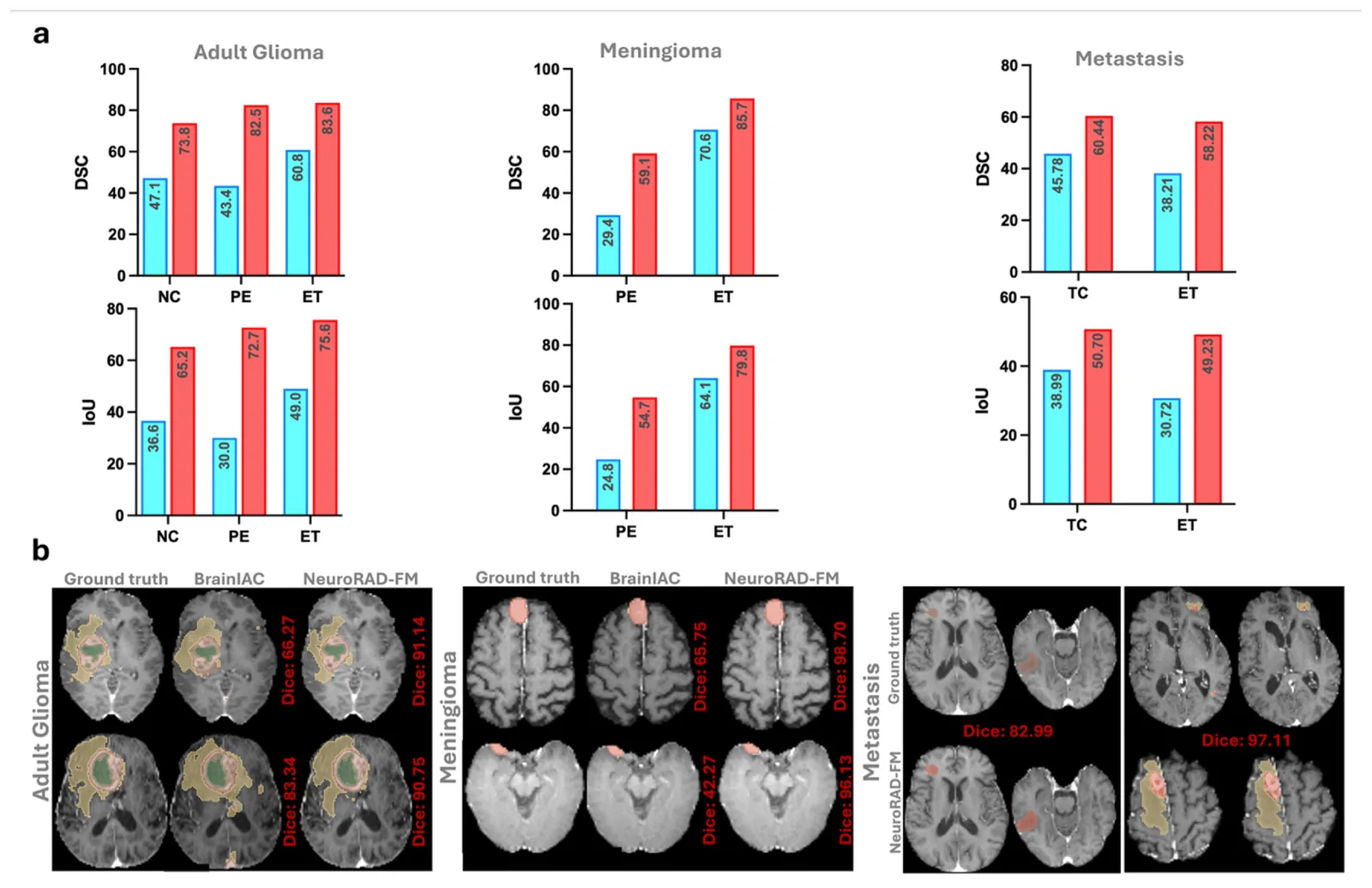
Cross-tumor segmentation performance of NeuroRAD-FM on the BraTS 2025 datasets. (a) Comparison of BrainIAC (sky blue) and NeuroRAD-FM (red) using DSC and IoU for adult glioma, meningioma, and brain metastasis segmentation. Evaluated compartments include NC, PE, and ET for glioma and meningioma segmentation, and TC and ET for metastasis segmentation, and (b) Representative contrast-enhanced T1-weighted images showing ground-truth annotations and segmentation predictions from BrainIAC and NeuroRAD-FM for adult gliomas, meningiomas, and brain metastases. Dice scores are displayed for each representative prediction. Green indicates NC, yellow indicates PE, and red indicates ET.

**Figure 4:**
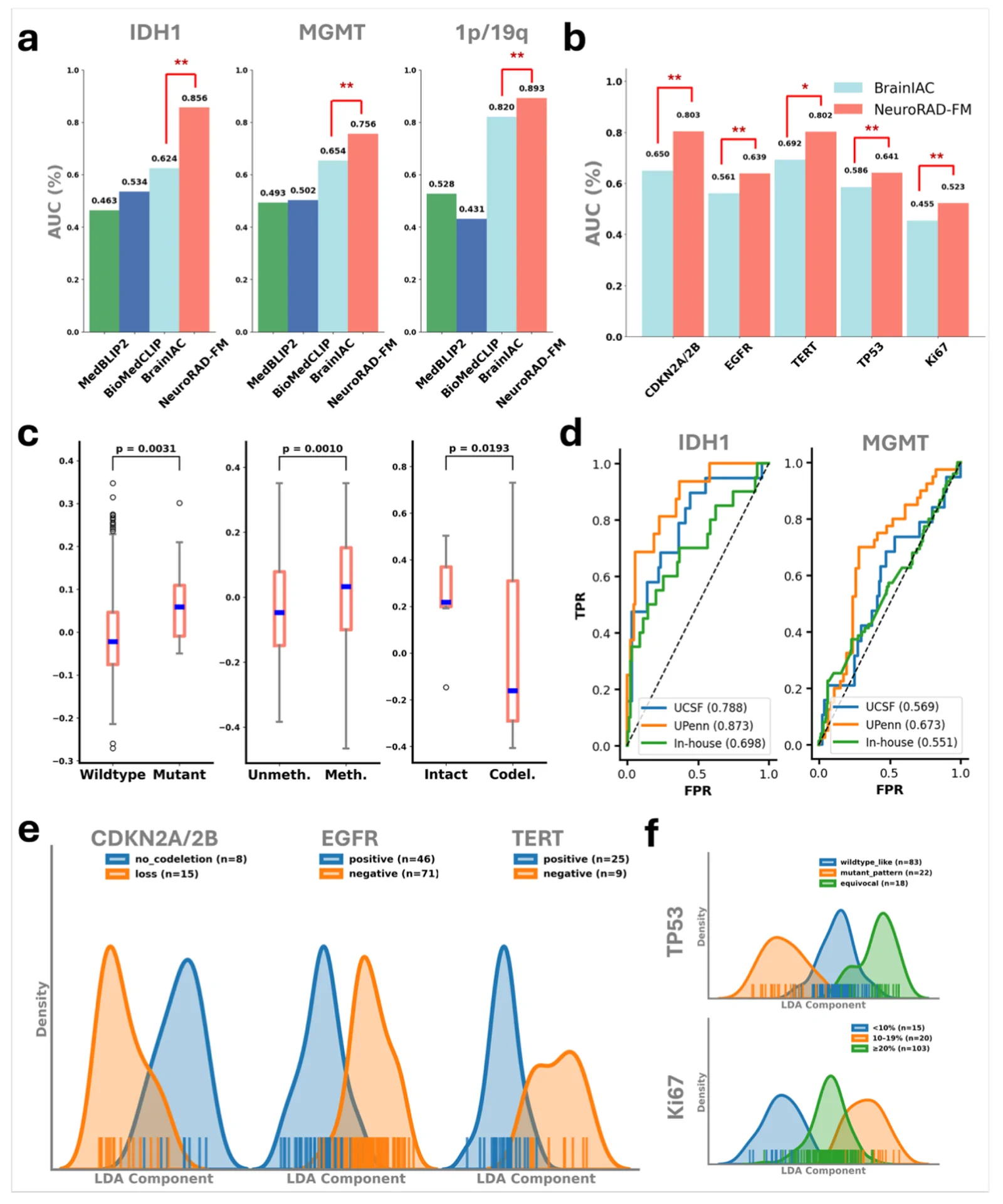
(a) Comparison of different baseline methods and NeuroRAD-FM for IDH1, MGMT prediction on a combined cohort (UPenn, UCSF, and In-house) and 1p/19q marker prediction for UCSF, and In-house cohorts, (b) Under-represented markers like CDKN2A/2B, EGFR, TERT, TP53, and Ki-67 prediction on the In-house cohort. (^∗∗^ indicates p < 0.01 and ^∗^ indicates p < 0.05), (c) Box plots of the NeuroRAD-FM feature embeddings for IDH (Wildtype vs Mutant), MGMT (Methylated vs Unmethylated) and 1p/19q (Intact vs co-deleted), (d) ROC-AUC curve for different cohorts (UPenn, UCSF and In-house), (e) 1D kernel density estimation (KDE) plots of the NeuroRAD-FM feature embeddings for binary classification of molecular marker CDKN2A/2B (no codeleted vs loss), EGFR and TERT (positive vs negative), and (f) 2D KDE plots for multiclass classification of TP53 (wildtype-like, equivocal and mutant-pattern) and Ki-67 index (<10%, 10–19% and ≥20%), illustrating class-wise distribution and separation in the learned feature space.

**Figure 5:**
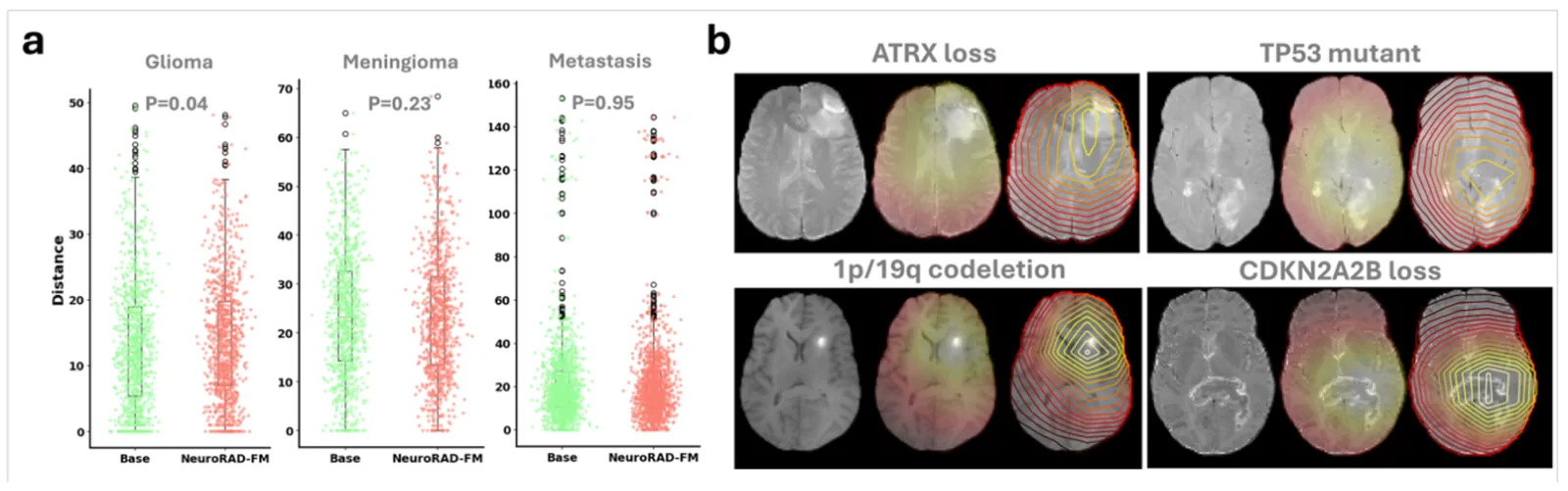
(a) Distance (in voxels) between the predicted attention centroid and ground-truth tumor centroid for the baseline model and NeuroRAD-FM across glioma, meningioma, and brain metastasis cohorts, and (b) Representative MR images with corresponding Grad-CAM heatmaps and contour visualizations demonstrating model attention for under-represented molecular alterations.

**Table 1:** Patient Demographics and Molecular Characteristics of the In-house Cohort (n = 292)

Characteristic	Category	N
Gender	Male	166
	Female	113
	NA	13
IDH mutation	Wildtype	255
	Mutant	25
	NA	12
MGMT promoter methylation	Methylated	86
	Unmethylated	172
	Partially Methylated	23
	NA	11
TERT	Positive	25
	Negative	9
EGFR	Positive	46
	Negative	71
CDKN2A/2B	Loss	15
	No codeletion	8
TP53	Wildtype-like	83
	Mutant pattern	22
	Equivocal	18
	Negative	14
Ki-67	≥ 20%	103
	10–19%	20
	< 10%	15

**Table 2:** External Cohort Demographics and Molecular Characteristics

Characteristic	Category	UCSF (n = 111)	UPenn (n = 95)
Sex/Gender	Male	68	62
	Female	43	33
IDH mutation	Wildtype	63	84
	Mutant/Mutated	48	11
MGMT promoter methylation	Methylated	80	39
	Unmethylated	19	45
	Not available/Indeterminate	12	11
1p/19q	Intact	96	–
	Co-deletion	9	–
	Relative co-deletion	6	–

**Table 3:** Survival analysis for UPenn, UCSF and the In-house cohorts. C-index is reported.

Dataset	BrainIAC	NeuroRAD-FM
UCSF	58.40 ± 17.13	66.42 ± 6.62
UPenn	66.86 ± 5.42	69.31 ± 3.21
In-house	59.11 ± 1.88	62.96 ± 5.11

## Data Availability

The datasets used for pre-training are publicly available from their respective sources. The clinical dataset from Columbia University Irving Medical Center (CUIMC) is an in-house dataset and is not publicly available because of institutional, privacy, and ethical restrictions. Access to the CUIMC dataset may be possible upon reasonable request and subject to institutional approval, applicable data-use agreements, and any required licensing or regulatory requirements.
